# FACTORS ASSOCIATED WITH RESIDENTIAL CARE COMMUNITIES THAT HAVE RESIDENTS VISITING EMERGENCY DEPARTMENTS

**DOI:** 10.1093/geroni/igz038.1879

**Published:** 2019-11-08

**Authors:** Amanuel Melekin, Christine Caffrey, Vincent Rome

**Affiliations:** Centers for Disease Control and Prevention/National Center for Health Statistics, Hyattsville, Maryland, United States

## Abstract

Emergency department (ED) visits are an important part of healthcare utilization. However, ED visits can be costly, lead to hospitalizations, and are sometimes unnecessary. Studies characterizing ED visits among long-term care settings have been largely focused on nursing homes where the unit of analysis is typically the resident. Facility- or community-level analyses describing residential care communities (RCCs) with ED visits are limited. Using RCCs as the unit of analysis, this study examines community-level factors associated with RCCs that have residents with ED visits. Community-level factors include ownership and chain affiliation, Medicaid participation, electronic health records use, service provision, nurse staffing, U.S. census region and metropolitan status. The study uses data from the 2016 National Study of Long-Term Care Providers conducted by the National Center for Health Statistics. In 2016, about 81% of RCCs had at least one resident visiting the ED in the past 90 days and around 19% of RCCs had no residents with ED visits in the past 90 days. Bivariate analyses indicated that ED visits varied by chain affiliation, ownership status, electronic health records use, and Medicaid participation. Logistic regression modeling to examine factors associated with whether or not RCCs had any residents with ED visits in the past 90 days will also be presented. Results may benefit efforts focused on implementing practices to reduce ED visits in RCCs.

